# To IMPRES or to EXPRES? Exploiting comparative judgments to measure and visualize implicit and explicit preferences

**DOI:** 10.1371/journal.pone.0191302

**Published:** 2018-01-19

**Authors:** Tom Everaert, Adriaan Spruyt, Jan De Houwer

**Affiliations:** Department of Experimental-Clinical and Health Psychology, Ghent University, Ghent, Belgium; Swansea University, UNITED KINGDOM

## Abstract

We introduce an adaptation of the affect misattribution procedure (AMP), called the implicit preference scale (IMPRES). Participants who complete the IMPRES indicate their preference for one of two, simultaneously presented Chinese ideographs. Each ideograph is preceded by a briefly presented prime stimulus that is irrelevant to the task. Participants are hypothesized to prefer the ideograph that is preceded by the prime they prefer. In the present research, the IMPRES was designed to capture racial attitudes (preferences for white versus black faces) and age-related attitudes (preferences for young versus old faces). Results suggest that (a) the reliability of the IMPRES is similar (or even better) than the reliability of the AMP and (b) that the IMPRES and the AMP correlate significantly. However, neither the AMP nor the IMPRES were found to predict attitude-related outcome behavior (i.e., the preparedness to donate money to a charity benefiting ethnic minorities vs. the elderly). Further research is thus necessary to establish the validity of the IMPRES. Finally, we demonstrated that, unlike the AMP, the IMPRES allows for an in-depth assessment of unanticipated response patterns and/or extreme observations using multidimensional scaling algorithms.

## Introduction

Preferences are vital determinants of behavior [1, 2]. Although these preferences are often assessed in a direct way via self-assessments, such *explicit measures* are known to be sensitive to social norms and/or privacy concerns. Moreover, some evaluations can be automatic in nature and might therefore be less accessible to conscious introspection. Consequently, innovative measures were developed that probe stimulus evaluations under automaticity conditions [3, 4]. Well-known examples are the evaluative priming task [5], the implicit association test (IAT, [6]), and the affect misattribution procedure (AMP, [7]). These so-called *implicit measures* have been the subject of abundant research showing predictive power over and above explicit measures [8–10].

In the AMP, for instance, participants are presented with a sequence of Chinese ideographs and asked to evaluate each of them as either “less pleasant” or “more pleasant” than the average Chinese ideograph. Each Chinese ideograph is preceded by the short presentation of a stimulus, defined as the prime, which is irrelevant to the task. Nevertheless, the prime presentation influences task performance in the sense that the likelihood of emitting a “more pleasant” response is higher when the prime is evaluated positively rather than negatively. It is common practice in the AMP and other implicit measures to use contrasting stimulus categories as primes, such as pictures of black faces and white faces. Task performance is typically converted into a score that reflects a person’s preference for one category over another. In the AMP, such scores are generally calculated by subtracting the proportion of positive responses given the presentation of prime from one prime category (e.g., black faces) from the proportion of positive responses after the presentation of a prime from other prime category (e.g., white faces). The validity of these scores has been attested in numerous studies in which preference scores were shown to predict explicit measures and relevant criterion behaviors, at least to some extent [9, 10].

Compared to other implicit measures, the AMP has several advantages that contributed substantially to its rising popularity. First, the AMP is relatively easy to implement because it requires only one factor to be randomized (i.e., the prime category) whereas other procedures typically require a more sophisticated randomization scheme with several factors. Second, the straightforward design of the AMP also facilitates its scoring (i.e., a simple subtraction of proportions) and, hence, the interpretation of the scores. Third, the use of proportions instead of reaction times for scoring makes the AMP an excellent tool to study preferences in populations that are known to respond relatively slowly and erratically, such as the elderly and clinically depressed patients. Fourth, measuring attitudes using evaluative judgments as opposed to reaction times may be more consistent with the theoretical structure of an attitude in that it naturally captures both the valence and the extremity of an evaluation [10]. Finally, the reliability and validity indices of the AMP scores are very competitive in comparison to those obtained with more complex implicit measures such as the evaluative priming paradigm.

The AMP also has some drawbacks, however. For instance, the task may feel somewhat unnatural. Participants are typically unfamiliar with Chinese ideographs and the use of “the average” Chinese ideograph as a point of reference may come across as somewhat odd and/or arbitrary. Another drawback pertains to the number of trials employed and the use of proportions as a metric. In general, implicit measures require some care in deciding how many trials are required to obtain a good estimate of one’s implicit evaluations. Presenting just a few trials might lead to unstable estimates, whereas presenting many trials might render the task too tedious. In the AMP, this problem is somewhat exacerbated because the AMP scores are simply proportions. For instance, if one were to calculate an AMP score on the basis of just 10 trials, only 11 different proportions of “more pleasant” responses can be observed (i.e., 0.0, 0.1, 0.2, …, 1.0). In such cases, proportions provide a relatively discrete and imprecise index, perhaps even more so than the mean speed of responding typically used for other implicit measurement procedures. The balance between the stability of the estimate and the effort required from the participant can therefore be more delicate in the AMP as compared to other implicit measures. Of course, one may note that the total number of AMP trials is typically well above 50 [10], but some authors did report that the total number of AMP trials can be as small as 18 (see [10], p. 682) or even 16 [11, 12]. Moreover, for some research questions, it may be essential to compute AMP scores for specific stimuli rather than stimulus categories. The number of observations that would be required to enable such item-level analysis would quickly rise above a workable level using a classic AMP.

To overcome these difficulties, we developed an adaptation of the AMP. In this procedure, called the implicit preference scale (hereafter referred to as the IMPRES), not one but two Chinese ideographs are presented simultaneously on the computer screen and participants are asked to indicate which of the two ideographs they prefer. Crucially, each of the two ideographs is preceded by the short presentation of a prime stimulus that is irrelevant to the task. We hypothesized that the preference for one ideograph over the other would be a function of the preference for one of the two primes. A relative preference score for one category over another is then indexed by the proportion of trials one which an ideograph preceded by a prime from one stimulus category (e.g., white faces) is preferred over an ideograph preceded by a prime of the other stimulus category (e.g., black faces).

We believe that this task is experienced as more natural as compared to the standard AMP task. After all, participants can readily compare two ideographs instead of having to rely on the abstract notion of “the average ideograph”. In addition, because two primes are presented in a single trial, any given number of trials captures more information in the IMPRES than the AMP. An IMPRES trial reflects the relative preference for one stimulus over another directly while at least two AMP trials would be necessary to achieve similar results. The estimates calculated from the IMPRES might therefore be more stable and precise compared to those calculated from the AMP. The extra prime presentations also allow for more fine-grained analyses at the level of the individual stimuli. Moreover, the calculation of the IMPRES score entails even less calculation as compared to the AMP, because one can simply use the proportion of trials on which an ideograph was chosen if it was preceded by a prime form a given prime category (e.g., white faces).

Another advantage of the IMPRES is the fact that the preference judgments are ideal for the use of multidimensional scaling (MDS) algorithms, thereby allowing for a straightforward visualization of implicit preferences and the identification of extreme subjects or stimuli and other peculiarities [13]. MDS algorithms create low-dimensional maps of the data by iteratively looking for a map with distances between entities that are as close as possible to their dissimilarities in the data. Although many MDS implementations exist, one method seems especially suited for the visualization of implicit preferences, i.e. multidimensional unfolding [14]. This algorithm allows for the visualization of both participants and stimuli in a single map. The distance between a participant and a stimulus in the map reflects that participant’s preference for that stimulus compared to the other stimuli. Short distances between a participant and a stimulus indicate stronger preferences for that stimulus as compared to long distances.

In this paper, we report the first study conducted with the IMPRES. The main motivation to conduct this study was to demonstrate that the IMPRES data are suited for a MDS modeling approach. The IMPRES used was designed to capture racial attitudes (preferences for white vs. black faces) and age-related attitudes (preferences for young vs. old faces). In a first step, the results obtained with the IMPRES were compared with the results obtained with the traditional AMP and explicit evaluative ratings. It might be argued, however, that it is somewhat difficult (or even incorrect) to compare the IMPRES (which is a relative measure) with explicit evaluative ratings of individual stimuli [15]. Accordingly, we also included an adaptation of the IMPRES in which no Chinese ideographs were presented. Instead, participants were simply presented, on each of a series of trials, with two stimuli and asked to indicate which stimulus they preferred over the other. We will refer to this procedure as the explicit preference scale or EXPRES. Importantly, as one can use the AMP to predict behavior [9, 10], a full comparison of the AMP and the IMPRES also requires an examination of the validity of these measures. Accordingly, we also included a behavioral outcome variable. More specifically, we asked participants to divide the monetary compensation for their participation (hypothetically) among themselves and two charities (i.e., one supporting ethnic minority groups and one supporting the elderly). Finally, and most importantly, both the IMPRES and the EXPRES data were subjected to an MDS analysis.

## Materials and methods

### Ethics statement

The procedures used in this manuscript were approved by the Ethics Committee of the Faculty of Psychology and Educational Sciences of Ghent University (approval number 2015/40). All participants gave (written) informed consent prior to participation.

### Participants

A sample of 53 students at Ghent University was selected via an online recruiting system. One may note that this sample size is well in line with the sample size used in other AMP studies. For example, in the seminal AMP paper [7], the sample size ranged between *N* = 33 and *N* = 55 across six experiments. Two participants did not supply demographic data. The remaining 51 participants (14 men) were on average 24 years old (*SD* = 6.58). They received €5 for their participation in the 30-minute study.

### Materials

The primes were 20 head-and-shoulders pictures of male faces that varied with regard to race (black vs. white) and age (young vs. old). There were five exemplars for each of the four possible combinations of both stimulus dimensions. These primes were selected from a set of 40 pictures that was used in the AMP studies by Gawronski and colleagues [16, 17]. The pictures were 203 pixels wide and 250 pixels high and displayed the faces against a blue background. The targets were 260 Chinese ideographs that were presented in black on a white background and measured 250 pixels by 250 pixels. A random pattern of black and white pixels that sized 256 pixels by 256 pixels was used as a backward mask for the targets.

The study included a small, 10-item questionnaire aimed to assess modern racism (MRS, [18]). Additionally, participants were asked to hypothetically divide the €5 they would earn among themselves and two charities supporting either ethnic minorities or the elderly. To increase the realism of the study, this allocation task was presented together with pictures of posters that pertained to these particular causes. The posters were taken from the website of ‘VZW Welzijnszorg’ (http://www.welzijnszorg.be), a local non-profit organization.

The experiment was programmed using Affect 4.0 [19] and presented with a desktop computer connected to a 17” flat screen monitor and a standard mouse and keyboard.

### Procedure

Participants were tested individually. They were seated in cubicles in front of the computer screen. After supplying some basic demographic data (age and gender), participants performed several implicit and explicit measures in a partially counterbalanced order (see below).

During the AMP task, participants were presented with 120 trials for which 120 unique Chinese ideographs were drawn randomly from the available set. Each of the 20 prime pictures was presented 6 times. During each AMP trial, participants were presented with, respectively, a fixation cross for 500 ms, a blank screen for 500 ms, a prime for 75 ms, a blank screen for 125 ms, a target for 100 ms, and a final backward mask until a response was emitted. Participants were asked to indicate whether the Chinese ideographs were “less pleasant” or “more pleasant” than the average Chinese ideograph, using the left ctrl key or the right ctrl key of the keyboard, respectively. Responses emitted after 1500 ms had elapsed resulted in a 750-ms feedback message that prompted the participant to respond faster.

During the IMPRES, each possible pairing of the prime stimuli was presented exactly once, resulting in 190 trials in total. Two of the 260 available Chinese ideographs were drawn randomly for each trial and were presented as targets. On each trial, participants were first presented with a 500-ms fixation cross in the center of the computer screen. Next, the two prime pictures were presented simultaneously for 150 ms, one left and one right of the central location. Note that we increased the presentation time of the primes relative to the AMP (i.e., 75 ms) to compensate for the fact that two prime stimuli were presented simultaneously. Next, after a blank screen of 150 ms, the two targets were presented simultaneously for 100 ms, again one left and one right of the central location. Finally, the two targets were replaced with two backward masks until participants emitted a response. Participants were asked to indicate which ideograph they preferred by pressing the ctrl key with the location that corresponded to that particular ideograph. Similar to the AMP, responses emitted after 1500 ms had elapsed triggered a feedback message prompting faster responding. Since every possible pair of primes was presented, some trials included primes from the same category (e.g. two black faces, or two old faces) that were uninformative with regard to the preference for that category. These trials were presented to facilitate analyses at the stimulus level, for instance, to identify differences in the relative preference for specific faces within the same stimulus category.

During the EXPRES, participants performed a task similar to the IMPRES. No Chinese ideographs were presented, however, and the stimulus pairs remained on the screen until a response was registered. Participants were asked to indicate which stimulus was preferred over the other by selecting the corresponding ctrl key. In contrast with the AMP or the IMPRES, no response deadline was imposed on the participants.

During the rating task, the 20 primes were presented sequentially alongside a 9-point rating scale that ranged from “highly unpleasant” to “highly pleasant”. Participants were asked to rate the pleasantness of each of the primes on this rating scale.

After performing these tasks, participants completed a computerized version of the modern racism scale (MRS) in which each of the 10 items was presented separately on screen alongside a 5-point likert scale. At the end of the experiment, participants were told they could pick up their payment of €5. Before receiving their payment, however, they were asked to hypothetically divide their money over three options: (1) money for themselves, (2) money for a charity fighting poverty in ethnic minority groups, and (3) money for charity fighting poverty amongst the elderly.

To prevent any influence from the explicit evaluation tasks on the performance in the implicit evaluation tasks, both the AMP and the IMPRES were presented before the rating task and the EXPRES. The order in which the two implicit tasks were presented and the order in which the two explicit tasks were presented, was counterbalanced between participants. The experiment always ended with the completion of the modern racism scale, followed by the charity question.

### Data analysis

The raw data of this experiment are available at https://doi.org/10.6084/m9.figshare.5135626.v1. The data were first subjected to standard data analyses. Analysis of the AMP data was conducted after excluding trials with latencies below 150 ms or above 1500 ms [17]. AMP scores were calculated for the race and age dimension by subtracting the proportion of “more pleasant” responses after a black or an old face from the proportion of “more pleasant” responses after a white or a young face, respectively.

In the IMPRES, 100 trials pitted faces of opposing categories against each other directly (i.e., black vs. white, or young vs. old). The data associated with these trials were used to assess the relative preferences for one race or age category over the other. A preference for white faces over black faces was indexed by the proportion of times that participants preferred an ideograph presented at the location of a white face. Likewise, a preference for young faces over old faces was indexed by the proportion of trials on which the preferred ideograph was preceded by a young face. The deviation of such a proportion from 0.50 is an indicator for the strength and direction of the relative implicit preference.

Similar preference scores were derived from the explicit measures. For the EXPRES, the proportion of times a white face was preferred over a black faces reflected the preference for white faces over black faces. The same logic was applied to obtain preference scores for young faces over old faces.

The pleasantness ratings were used to calculate more conventional explicit preference scores. To obtain an explicit relative preference score for race, the mean pleasantness rating for black faces was subtracted from the mean preference rating for white faces. The explicit relative preference score for age was calculated in the same manner. The scores on the MRS were calculated by summing the scores of all items after reversing the scales of the items that were formulated in a negative manner.

The reliability of the preference scores on the AMP, IMPRES, EXPRES, and the rating task was calculated by performing 10,000 runs in which the data of each participant were divided randomly into two subsets. In each run, preference scores were calculated for each subset and subsequently correlated. The 10,000 correlations were then averaged and Spearman-Brown corrected to compensate for the loss of power associated with the use of smaller subsets.

To examine the associations between the different measures, straightforward correlational analyses were performed. To investigate the relation between charity donations and the preference measures, multivariate linear regressions were run in which the donations to the three possible causes (charity for minorities, charity for elderly, or the participant) were regressed on each score separately. This analysis was performed to take into account the covariance between the charity donations, as giving more to one cause necessarily implied giving less to the other two causes.

Finally, the IMPRES and EXPRES data were subjected to multidimensional scaling. A matrix that contained the number of times that each face picture was preferred by each participant was subjected to a metric multidimensional unfolding model in R using the SMACOF package [20]. This model yielded a map in which the distance between a participant and a face picture reflected that participant’s preference for that picture. The map was obtained by iteratively improving an initial map until improvement was negligible or until a maximum number of iterations was reached. The fit criterion that was minimized to attain the eventual solution is called the *stress*, which expresses the error of the map on a scale of 0 to 1. The dimensionality of the final map was decided on by visual inspection of the solution, by inspection of the stress of the solution, and by assessing whether or not the stress improved substantially by increasing the dimensionality of the solution.

The resulting representations were rotated so that the principal axes corresponded as good as possible to the two underlying dimensions of the primes (i.e., race and age). The principal axes are orthogonal and might therefore not align with the race and age dimension completely. If the preferences for race and age are correlated, however, the axes corresponding to these features would not be perpendicular to one another. Accordingly, we added additional axes that resulted in a maximal separation of the stimuli along the race dimension as well as the age dimension. The cosine of the angle between the two axes thus reflects the correlation of the race preference scores with the age preference scores. The coordinates of the axes were derived from a bias-reduced logistic regression [21] in which the features of interest (black vs. white or old vs. young) were regressed on the coordinates of the stimuli in the representation.

## Results

After deleting trials with response latencies that were identified as outliers (4.59%), AMP scores were calculated for the two prime dimensions (i.e., race and age). The mean AMP score did not differ significantly from zero, neither for race, *M* = -0.036, *s* = 0.163, *t*(52) = -1.61, *p* = 0.113, nor for age, *M* = 0.021, *s* = 0.108, *t*(52) = 1.44, *p* = 0.157. The reliability of the race AMP scores was reasonably high, *r* = .69, *t*(52) = 6.82,*p* < .0001, but the reliability of the age AMP scores was low, *r* = .28, *t*(52) = 2.11, *p* = .010.

In the IMPRES, the proportion with which white faces were (implicitly) preferred over black faces did not differ significantly from 0.50, *M* = 0.49, *s* = 0.09, *t*(52) = -1.13, *p* = 0.267. Young faces, however, were preferred over old faces, *M* = 0.52, *s* = 0.08, *t*(52) = 2.18, *p* = 0.003. The reliability coefficients for both measures were reasonably high for both race, *r* = .69, *t*(52) = 6.73, *p* < .0001, and age, *r* = .63, *t*(52) = 5.85, *p* < .0001.

The EXPRES data revealed a significant tendency to (explicitly) prefer white faces over black faces, *M* = 0.58, *s* = 0.20, *t*(52) = 3.07, *p* = 0.003, and a significant tendency to prefer young faces over old faces, *M* = 0.57, *s* = 0.20, *t*(52) = 2.76, *p* = 0.008. The reliability of both EXPRES scores was very high, *r* = .95, *t*(52) = 21.20, *p* < .0001, and *r* = .95, *t*(52) = 20.86, *p* < .0001, respectively for race and age.

The mean evaluative ratings showed significant deviations from zero with regard to race, *M* = 0.374, *s* = 1.137, *t*(52) = 2.392, *p* = 0.020, but not with regard to age, *M* = 0.132, *s* = 1.267, *t*(52) = 0.759, *p* = 0.452. The reliability of the rating scores was moderate to high, *r* = .59, *t*(52) = 5.21, *p* < .0001, for race, and *r* = .67, *t*(52) = 6.49, *p* < .0001, for age. The mean MRS score did not differ significantly from the theoretical midpoint of 25, *M* = 25.87, *s* = 5.74, *t*(52) = 1.010, *p* = 0.276. When asked to hypothetically divide their payment of €5 amongst two charities and themselves, participants allocated on average €2.43 (*SD* = €1.60) to a charity benefiting racial minorities, €1.36 (*SD* = €1.18) to a charity benefiting the elderly, and €1.14 (*SD* = €0.91) to themselves.

The correlations between the scores are displayed in Table 1. The correlations between the preference scores for race are presented in the top left. Several significant correlations were observed. First, a significant association was observed between the AMP and the IMPRES. Second, significant associations were observed between the rating scores, the EXPRES scores, and the Modern Racism scale. Especially the correlation between the rating scores and the EXPRES scores proved to be highly significant. The IMPRES did not correlate significantly with any of the explicit measures whereas the AMP was found to be related significantly with both the EXPRES score and the rating score. Multivariate linear regressions of the three charity variables on the race preference measures showed no significant associations between charity donations and the AMP score, *F* < 1, the IMPRES score, *F* < 1, or the EXPRES score, *F*(3, 48) = 1.60, *p* = .20. The regression of charity donations on the rating scores, however, did reach significance, *F*(3, 48) = 3.00, *p* = .040, *η*^*2*^ = .16, suggesting a tendency for participants with a high relative preference for white faces over black faces to donate less to a charity for racial minorities and themselves, but more to a charity for the elderly. An additional multivariate regression of charity donations on the MRS showed a marginally significant association, *F*(3, 48) = 2.54, *p* = .067, *η*^*2*^ = .14. Similar to the rating scores, the MRS was associated with donating less to one’s self, but not more to a charity for racial minorities.

**Table 1:** Pearson rank correlations between the conventional attitude and behavioral measures.

	Race—AMP	Race—IMPRES	Race—EXPRES	Race—Rating	Modern Racism	Race—Charity	Age—AMP	Age—IMPRES	Age—EXPRES	Age—Rating	Age—Charity
Race—IMPRES	.35**										
Race—EXPRES	.33**	.11									
Race—Rating	.29**	.01	.84***								
Modern Racism	.18	.13	.34**	.31**							
Race—Charity	-.04	.02	-.10	-.13	.11						
Age—AMP	.03	.26*	-.02	-.01	.06	.21					
Age—IMPRES	.03	-.03	-.01	-.03	.07	-.03	.15				
Age—EXPRES	.16	.13	.10	.20	.07	-.05	.14	.21			
Age—Rating	.11	.19	.09	.10	.04	.05	.26*	.23	.82***		
Age—Charity	.21	.05	.12	.16	-.06	-.61***	-.22	-.08	.04	.04	
Self—Charity	.06	.05	-.15	-.20	-.34**	-.49***	-.16	.02	-.11	-.09	.54***

* = *p* < .10,

** = *p* < .05,

*** = *p* < .001

The correlations between the preference scores for age are presented in the bottom right of Table 1. Only one correlation reached significance within this subset, suggesting a strong, positive association between the rating score and the EXPRES score. A marginal trend also hinted at a positive association between the AMP and the rating scores, but no other correlation approached significance. Multivariate linear models of the charity donations on the age preference scores showed no significant associations, all *F*s < 1.

None of the correlations between the measures of race preference and age preference reached significance. The charity donations correlated significantly with one another. This observation was to be expected as giving more to one charity necessarily implied that less money was available for another charity. This strong interconnection further stresses the need for multivariate analysis techniques over univariate correlation analyses for this measure.

**Fig 1:**
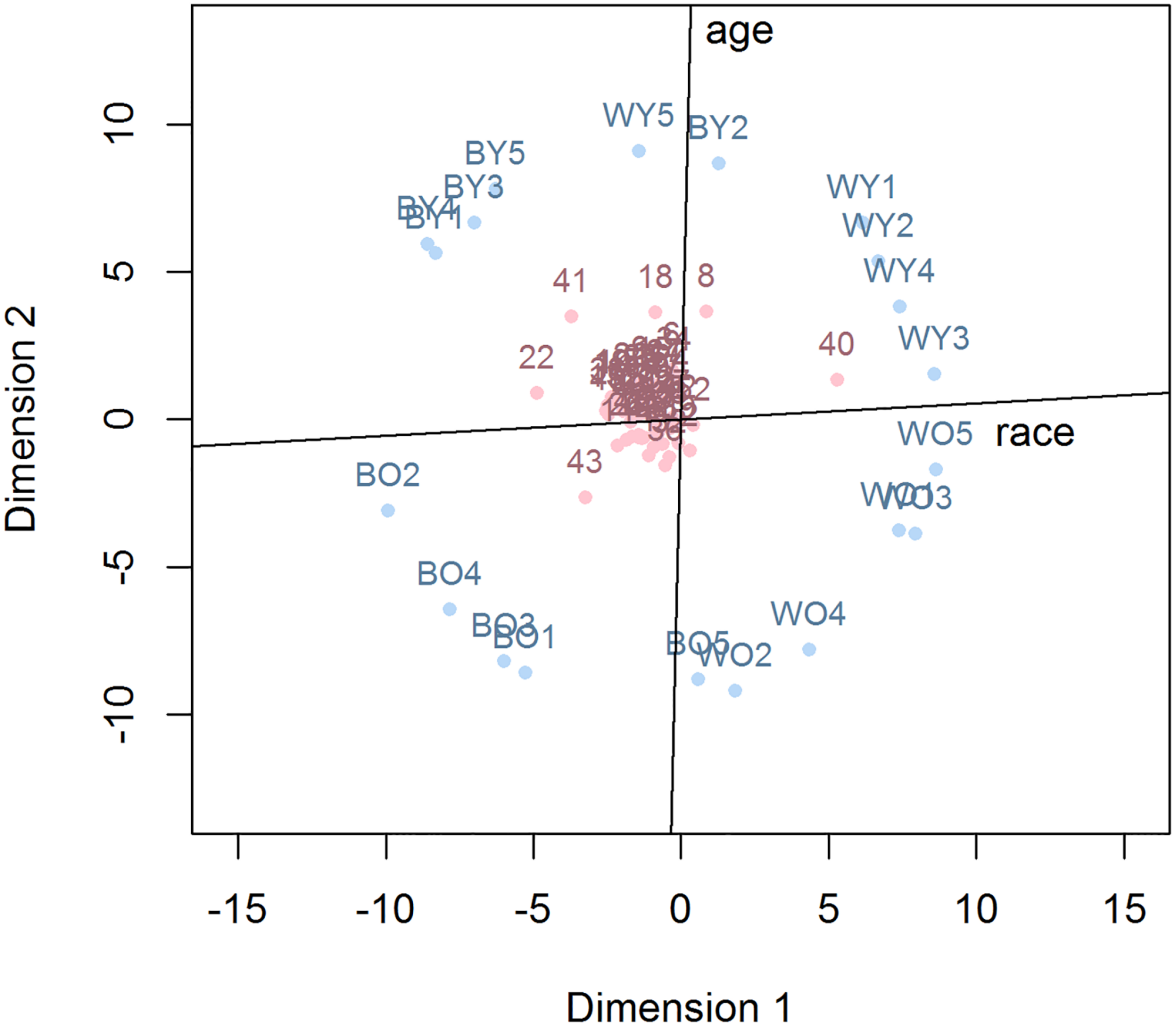
Visualization of the IMPRES data with additional axes that maximally separate the race and the age of the primes. Points corresponding to the prime stimuli are presented in blue. The first letter indicates race (B vs. W, referring to black vs. white faces, respectively). The second letter indicates age (O vs. Y, referring to old vs. young faces, respectively). Points corresponding to the participants are presented in red.

Next, we subjected he IMPRES and EXPRES data to a multidimensional unfolding analysis. A two-dimensional solution of the IMPRES data yielded a two-dimensional solution with a minimal stress-value of 0.22 after 2083 iterations. Such a stress-level is rather high and might be indicative of two different scenarios. Either the two-dimensional solution failed to summarize the data in an adequate fashion or the data were simply somewhat noisy. We therefore also fitted a 3-dimensional solution to the data, resulting in a marginal reduction of the stress-level (i.e., after 1952 iterations, this model yielded a minimal stress-level of .20). Accordingly, a two-dimensional solution seems to capture the variation in the data quite well. In line with this conclusion, a quick glance at Fig 1 reveals a good separation between black faces (left) and white faces (right) and between young faces (top) and old faces (bottom). The points corresponding to the participants are organized with regard to their preference toward the faces in the experiment. For instance, a participant with a preference for white faces over black faces will be located closer to the white faces than the black faces. Fig 1 also reveals that some participants are extreme responders in the sense that they fall outside the main participant cluster in the center. Participant 40, for instance, responded in a manner suggesting a strong preference for white faces whereas the responses of participant 22 suggested a strong preference for black faces. It may be noted that exclusion of these extreme responders eliminated the correlation between the IMPRES scores and the AMP scores for the race dimension, *r* = -0.03, *p* = 0.85, whereas other correlations were largely unchanged. No strong outliers were present at the level of the primes, although stimulus WY5 is represented more on the left than the other white faces and stimulus BY2 is located more to the right than the other black faces. Although the principal axes coincided visually with race and age, bias-reduced logistic regressions were used to project additional axes on the map that maximally separated different races and ages. As expected, in the optimal solution, these axes were slightly correlated, *r* = .08.

Although the points corresponding to the participants are clustered very tightly around the center, their locations might still reflect meaningful inter-individual differences. To investigate this, preference scores were derived from the participant locations by orthogonally projecting the points on the axes that maximally separated race and age. Correlations of these scores with the other measures showed significant associations with the aforementioned IMPRES scores for race, *r* = .94, *p* < .0001, and age, *r* = .97, *p* < .0001, respectively. The participant locations are therefore informative and not the result of a degenerate multidimensional scaling solution.

**Fig 2:**
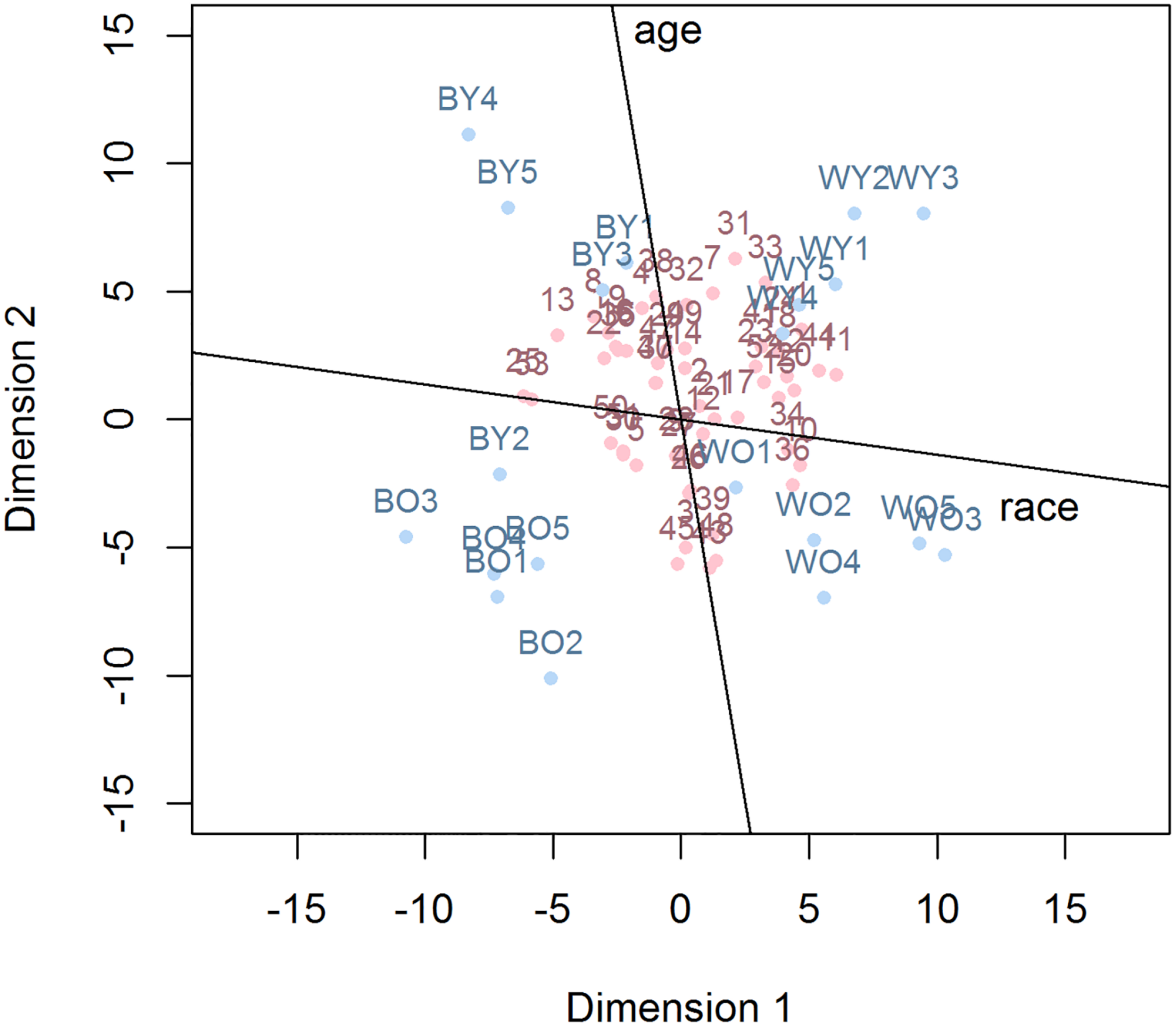
Multidimensional unfolding solution for the EXPRES data with additional axes for race and age. Points corresponding to the prime stimuli are presented in blue. The first letter indicates race (B vs. W, referring to black vs. white faces, respectively). The second letter indicates age (O vs. Y, referring to old vs. young faces, respectively). Points corresponding to the participants are presented in red.

Finally, to visualize the more explicit relative preferences, we also subjected the EXPRES data to multidimensional unfolding analysis. The resulting map had a minimal stress level that was somewhat higher than the one obtained with the IMPRES, stress = .34. Adding an extra dimension to the map did not result in substantial increase of the fit, stress = .31, which suggested that a two-dimensional solution captured the data quite well. Instead, the high stress seems to have resulted from noise and idiosyncrasies regarding the response strategies used by the participants. As can be seen in in Fig 2, the map of the EXPRES data reveals many more points that are scattered around as compared to the map of the IMPRES data. This difference further attests to the many idiosyncrasies involved in explicit decision making.

Here too, some peculiarities can be noted. The points associated with the primes tended to cluster together, suggesting that participants were evaluating the primes with regard to their category rather than their identities. The prime BY2 tends to be closer to the category of black old men than to the category of black young men, and might therefore be unrepresentative of its category. The cluster of participants is spread out more compared to the IMPRES representation, with more variation on the race dimension than on the age dimension, showing stronger inter-individual differences with regard to racial preferences than age preferences. The overlaid axes corresponding to the race and age dimension are clearly non-orthogonal, *r* = -.30, and show a tendency for explicit preferences towards whites to co-occur with explicit preferences towards the elderly.

## Discussion

In this paper, we introduced the IMPRES measure, which is a natural extension of the AMP. In the AMP, a trial consists of the presentation of a single Chinese ideograph that is preceded by the presentation of a single prime stimulus. In the IMPRES, two Chinese ideographs are presented simultaneously, each preceded by the short presentation of a prime stimulus. Participants are asked to indicate which of the two ideographs they prefer rather than judging their valence through an arbitrary comparison with the “average” Chinese ideograph.

The ease with which IMPRES preference scores can be derived is an advantage relative to the traditional AMP and does not seem to come at a cost in terms of reliability. In comparison to the reliability of the AMP, the reliability of the IMPRES measure was equally good (the race scores) or even better (the age scores). As this is the very first study in which the IMPRES was used, some caution is order when evaluating this data pattern. If, however, further research would show that the reliability of the IMPRES is on average better than the reliability of the AMP, it could be worthwhile to examine the precise reason(s) for this observation. As a first possibility, one could hypothesize that the pairwise presentation of the prime stimuli in the IMPRES increases the saliency of the contrasting prime categories. As an alternative hypothesis, one could argue that participants simply have more time to analyze the primes in the IMPRES because of the use of longer presentation times as compared to the AMP (150 ms vs. 75 ms, respectively).

The results suggested that the IMPRES scores were associated positively with the AMP scores, although statistical significance was observed for the race dimension only. The weak correlations between various preference measures of age probably resulted from the fact that (a) there was little variation in terms of age preference in our sample and (b) the reliability of the AMP was quite low for the age dimension [22].

We also included an adaptation of the IMPRES to capture explicit relative preferences. In the EXPRES, participants simply indicate which of two stimuli they prefer. Explicit preference scores are derived in the same manner as IMPRES scores. The strong similarity in methodology and scoring allows for a clearer comparison of implicit and explicit preference scores relative to the comparisons generally performed in attitude research [15]. Often, scores are compared that stem from widely different paradigms. It is common practice, for instance, to compare the implicit preference scores derived from an IAT with explicit preference scores derived from Likert-type rating scales. The comparison between such implicit and explicit preference scores can be hampered substantially because the measures differ in more aspects than the underlying construct they are purported to measure. A difference between the scores can therefore be attributed to other, methodological differences instead of the implicit/explicit distinction [15]. The EXPRES was found to correlate strongly with the other explicit preference measures, both for race and age.

To investigate the association of implicit and explicit preference measures with more natural behavior, we asked participants to distribute (hypothetically) the money they earned for their participation among a good cause benefiting ethnic minorities, a good cause benefiting the elderly, or themselves. A significant association with donating behavior was observed only for the explicit rating scores whereas a marginal association was found with the MRS. Both effects seemed to suggest that a preference for whites over blacks is associated with keeping less money in the pocket and donating more to charities related to the elderly than to ethnic minorities. Social desirability concerns might have prompted participants with stronger racial preferences to allocate more money to the charity benefiting the elderly. Because none of the implicit measures was found to have significant predictive abilities, the current data do not allow for strong conclusions with regard to the potential added value of the IMPRES as compared to the AMP. Nevertheless, by introducing the IMPRES, our work does set the stage for future research in which the merits of the IMPRES can be examined further.

In addition, it is an important advantage of the IMPRES that the IMPRES data are suited to be subjected to multidimensional scaling, a method that allows for an insightful visualization of implicit preferences in a low-dimensional space. This method is very convenient for exploratory research in implicit attitudes, as the resulting representations can be inspected easily for interesting data patterns and outliers. The multidimensional unfolding model [14] was fitted to the data to represent preferences as distances in a low-dimensional map. Outliers were detected quickly and other peculiarities regarding the specific stimuli were observed. This method might therefore prove valuable in the researcher’s arsenal of data inspection tools, especially in research where the detection of influential observations is warranted. Moreover, the use of multivariate techniques, such as multidimensional scaling, facilitates the detection of atypical observations in the space defined by multiple variables. Such observations can go undetected when using purely univariate inspection methods. Suppose, for instance, that the implicit preferences regarding race and age were highly correlated in a positive way. In such a situation, a participant with a large preference for a particular race but a low preference for a particular age would clearly be atypical, but this might go undetected if inspection is limited to race preferences and age preferences separately. The detection of outliers does not warrant the exclusion of a particular stimulus or observation from further analyses, however. Such measures should be taken only if the outlying values are the result of an error or if they pose a strong influence on the outcome of the analysis.

Additional axes were added to the plot to maximize the separation between the stimulus categories along each dimension. These axes were found by performing a regression of the feature of interest (i.e. race or age) on the coordinates of the stimuli. The parameters from the resulting regression equation can be used to project the axis onto the MDS solution. In the current study, bias-reduced logistic regression was used to project the categorical stimulus features onto the solution. Similar analyses can be performed with continuous features, however. In this case, the orientation of the axes is not determined by the parameter estimates of a logistic regression, but by the parameter estimates of a linear regression of the continuous feature on the coordinates of the prime. For instance, one could ask participants to rate the faces on various features, such as trustworthiness, physical attractiveness, friendliness, and so on. The axes related to these features could then be added to the plot, which would allow for the identification of participants who, for example, put more emphasis on trustworthiness or friendliness. The goodness-of-fit of the linear regression (e.g., the *R*^2^), could be used to assess to what extent the dimension is present in the solution and to what extent participants use this dimension in their judgments. In addition to adding continuous axes to the plot, clustering analyses could be performed to identify groups of participants or stimuli with similar characteristics.

In sum, as an extension of the AMP, we developed a new implicit measure that captures the preference for one stimulus (or stimulus category) over another. The method yielded acceptable reliability estimates and allows for insightful visualizations of implicit preferences, a unique feature that can aid data inspection and exploration.

## References

[1] Allport GW. Attitudes In: Murchison C, editor. A handbook of social psychology. Worcester, MA: Clark University Press.; 1935 p. 798–844.

[2] Martin I, Levy AB. Evaluative conditioning. Advances in Behaviour Research and Therapy. 1978;1:57–101.

[3] De Houwer J, Teige-Mocigemba S, Spruyt A, Moors A. Implicit measures: A normative analysis and review. Psychol Bull. 2009;135(3):347–68. doi: 10.1037/a0014211 19379018 10.1037/a0014211

[4] Nosek BA, Hawkins CB, Frazier RS. Implicit social cognition: from measures to mechanisms. Trends Cogn Sci. 2011;15(4):152–9. doi: 10.1016/j.tics.2011.01.005 21376657 10.1016/j.tics.2011.01.005 PMC3073696

[5] Fazio RH, Sanbonmatsu DM, Powell MC, Kardes FR. On the automatic activation of attitudes. J Pers Soc Psychol. 1986;50(2):229–38. doi: 10.1037//0022-3514.50.2.229 3701576 10.1037//0022-3514.50.2.229

[6] Greenwald AG, McGhee DE, Schwartz JLK. Measuring individual differences in implicit cognition: The implicit association test. J Pers Soc Psychol. 1998;74(6):1464–80. 9654756 10.1037//0022-3514.74.6.1464

[7] Payne BK, Cheng CM, Govorun O, Stewart BD. An inkblot for attitudes: Affect misattribution as implicit measurement. J Pers Soc Psychol. 2005;89(3):277–93. doi: 10.1037/0022-3514.89.3.277 16248714 10.1037/0022-3514.89.3.277

[8] Greenwald AG, Poehlman TA, Uhlmann EL, Banaji MR. Understanding and using the implicit association test: III. Meta-analysis of predictive validity. J Pers Soc Psychol. 2009;97(1):17–41. doi: 10.1037/a0015575 19586237 10.1037/a0015575

[9] Cameron CD, Brown-Iannuzzi JL, Payne BK. Sequential priming measures of implicit social cognition: A meta-analysis of associations with behavior and explicit attitudes. Pers Soc Psychol Rev. 2012;16(4):330–50. doi: 10.1177/1088868312440047 22490976 10.1177/1088868312440047

[10] Payne BK, Lundberg KB. The affect misattribution procedure: Ten years of evidence on reliability, validity, and mechanisms. Social and Personality Psychology Compass. 2014; (8):672–86. doi: 10.1111/spc3.12148

[11] Vanaelst J, Spruyt A, De Houwer J. How to modify (implicit) evaluations of fear-related stimuli: effects of feature-specific attention allocation. Front Psychol. 2016;7:ARTN 717 doi: 10.3389/fpsyg.2016.00717 27242626 10.3389/fpsyg.2016.00717 PMC4865498

[12] Vanaelst J, Spruyt A, Everaert T, De Houwer J. Extinction of likes and dislikes: Effects of feature-specific attention allocation. Cognition and Emotion. in press. doi: 10.1080/02699931.2016.1250724 27838952 10.1080/02699931.2016.1250724

[13] Borg I, Groenen PJF. Modern multidimensional scaling: Theory and applications. New York: Springer; 2005.

[14] De Leeuw J. Multidimensional unfolding In: Everitt BS, Howell DC, editors. Encyclopedia of statistics in behavioral science. 3 New York: Wiley; 2005 p. 1289–94.

[15] Payne BK, Burkley MA, Stokes MB. Why do implicit and explicit attitude tests diverge? The role of structural fit. J Pers Soc Psychol. 2008;94(1):16–31. doi: 10.1037/0022-3514.94.1.16 18179315 10.1037/0022-3514.94.1.16

[16] Gawronski B, Ye Y. Prevention of intention invention in the affect misattribution procedure. Soc Psychol Pers Sci. 2015;6(1):101–8. doi: 10.1177/1948550614543029

[17] Gawronski B, Cunningham WA, Lebel EP, Deutsch R. Attentional influences on affective priming: Does categorisation influence spontaneous evaluations of multiply categorisable objects? Cognition Emotion. 2010;24(6):1008–25. doi: 10.1080/02699930903112712

[18] McConahay JB. Modern racism, ambivalence, and the modern racism scale In: Dovidio JF, Gaertner SL, editors. Prejudice, discrimination, and racism. San Diego, CA, US: Academic Press; 1986 p. 91–125.

[19] Spruyt A, Clarysse J, Vansteenwegen D, Baeyens F, Hermans D. Affect 4.0 a free software package for implementing psychological and psychophysiological experiments. Exp Psychol. 2010;57(1):36–45. doi: 10.1027/1618-3169/a000005 20178962 10.1027/1618-3169/a000005

[20] De Leeuw J, Mair P. Multidimensional scaling using majorization: SMACOF in R. Journal of Statistical Software. 2009;31(3):1–30. doi: 10.18637/jss.v031.i03

[21] Firth D. Bias reduction of maximum likelihood estimates. Biometrika. 1993;80(1):27–38. doi: 10.1093/biomet/80.1.27

[22] De Schryver M, Hughes S, Rosseel Y, De Houwer J. Unreliable yet still replicable: A comment on LeBel and Paunonen (2011). Front Psychol. 2016;6:ARTN 2039. 26793150 10.3389/fpsyg.2015.02039 PMC4710742

